# Influence of *Saccharomyces* and non-*Saccharomyces* Yeasts in the Formation of Pyranoanthocyanins and Polymeric Pigments during Red Wine Making

**DOI:** 10.3390/molecules24244490

**Published:** 2019-12-08

**Authors:** Antonio Morata, Carlos Escott, Iris Loira, Juan Manuel Del Fresno, Carmen González, Jose Antonio Suárez-Lepe

**Affiliations:** enotecUPM, Universidad Politécnica de Madrid, Madrid 28040, Spain; carlos.escott@gmail.com (C.E.); iris.loira@upm.es (I.L.); juanmanuel.delfresno@upm.es (J.M.D.F.); carmen.gchamorro@upm.es (C.G.); joseantonio.suarez.lepe@upm.es (J.A.S.-L.)

**Keywords:** red wines, yeasts, non-*Saccharomyces*, pyranoanthocyanins, vitisins, vinylphenolic adducts, polymeric pigments

## Abstract

Yeast are able to modulate many sensory parameters of wines during red must fermentation. The effect on color and on the formation of derived pigments during fermentation has been studied thoroughly since the 90s. Yeast can increase grape anthocyanin’s color by acidification by hyperchromic effect (increase of flavylium molecules). Recent studies with non-*Saccharomyces* species, as *Lachancea thermotolerans,* described the intense effect of some strains on anthocyanin’s color, and subsequent, stability, by strongly reducing wine’s pH during fermentation. Moreover, selected yeast strains of *Saccharomyces* have been shown to release metabolites such as pyruvic acid or acetaldehyde that promote the formation of vitisin A and B pyranoanthocyanins during must fermentation. *Schizosaccharomyces pombe,* because of its specific metabolism, can produce higher concentrations of pyruvate, which enhances the formation of vitisin A-type derivatives. The hydroxycinnamate decarboxylase activity that some *Saccharomyces* strains express during fermentation also promotes the formation of vinylphenolic derivatives. Some non-*Saccharomyces* species, such as *S. pombe* or *P. guilliermondii* can also improve the production of these derivatives compared to selected strains of *Saccharomyces cerevisiae*. Lastly, some yeasts are also able to modulate the formations of polymeric pigments between grape anthocyanins and flavonoids, such as catechins and procyanidins.

## 1. Color and Wine Freshness

Even though freshness is more connected with mouth taste—mainly acidity, but also fruitiness and the absence of winey smells (fusel alcohols-like aroma) which produce flat wines (lack of fruity aroma and acidity) [1]—there is also a correspondence between red bluish color and youngness. Purple colors transmit a taster the visual perception of a young wine with the absence of oxidations and ageing, so this predisposes the taster to perceive the wine as younger and fruitier. Therefore, the preservation of blue hues in the red color of wines is a way to show a fresher wine. Similar perceptual illusion has been described for wines between color and odor [2], when red-dyed white wines were described as red wines by tasters. Several strategies in which *Saccharomyces* and non-*Saccharomyces* yeasts can participate as potential bio-tools to promote this perception, among them [3]: the biological acidification [4,5], the formation of stable red bluish pigments [6,7,8], the low adsorption of grape anthocyanins in yeast cell walls [9,10] and the release of natural antioxidant compounds, such as glutathione (GSH) during the reductive lees ageing [11,12], are some of them. Other possibilities are the addition of protective compounds such as phenols or the enhancement of copigmentation processes which also open up interesting opportunities [13].

## 2. Anthocyanins and Pyranoanthocyanins: Vitisins and Vinylphenolics Adducts

**Figure 1:**
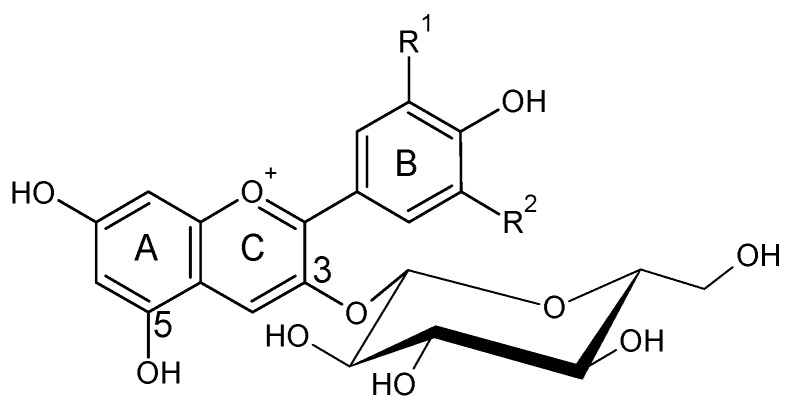
Flavylium form of the structures of *Vitis vinifera* anthocyanidin monoglucosides. Delphinidin R^1^: –OH; R^2^: –OH; cyanidin R^1^: –OH; R^2^: –H; petunidin R^1^: –OCH_3_; R^2^: –OH, peonidin R^1^: –OCH_3_; R^2^: –H; malvidin R^1^: –OCH_3_; R^2^: –OCH_3_.

**Figure 2:**
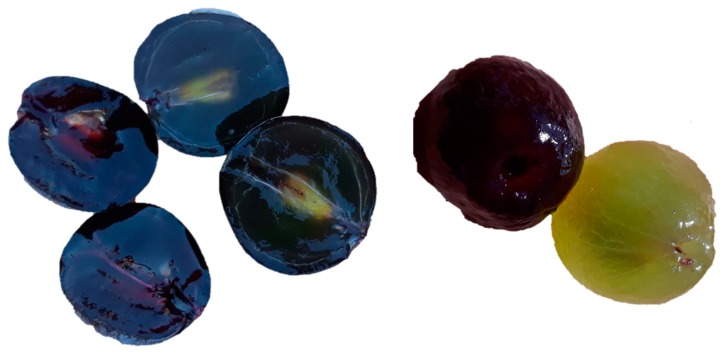
Grapes with and without pigmented pulp. On the left grapes cut in half, and on the right peeled berries from varieties Garnacha and Garnacha Tintorera (also known as Alicante Bouschet) with red pulp.

Anthocyanins are flavonoids (C6-C3-C6) with a pyrilium ring responsible for the absorption in visible spectra (Figure 1). Their properties have been defined in several articles and book chapters [14,15,16,17,18,19,20]. Anthocyanins are located in the skins of most varieties of *Vitis vinifera* L.; however, a few also have anthocyanins in the pulp (Figure 2). In order to extract the anthocyanins in the juice, it is necessary to macerate the skins. This process is done in the fermentation tank during red winemaking by favoring the contact between the solid parts of the grape and the must. Usually, around 17 anthocyanins and acylated anthocyanins can be found in significant amounts in grape juices from *V. vinifera* L., ranging from several hundreds to few milligrams per liter, all of them as 3-*O*-glucosides [21]. The aglycone is called anthocyanidin. The main are the single 3-*O*-glucosides of delphinidin, cyanidin, petunidin, peonidin and malvidin, the major grape anthocyanin.

Anthocyanin structures differ in the hydroxylation or methoxilation patterns in the B ring which affect the color, being red–bluer at higher methoxilation; their color is also pH-dependent (Figure 1, [22]). The methoxilation degree makes the anthocyanin less polar, but also increases its stability. In most of *Vitis vinifera* L. varieties, it is also typical to find the acylated derivatives of the previous five anthocyanins in position 6 of the glucose molecule. These acylated derivatives can be formed mainly with acetic, *p*-coumaric and caffeic acids, giving rise to the respective acetylated, *p*-coumaroyl and caffeoyl derivatives [23]. Some varieties as Pinot noir have strongly inhibited the formation of acylated derivatives, and usually only the five 3-*O*-glucosides can be found [24,25].

## 3. Vitisins

**Figure 3:**
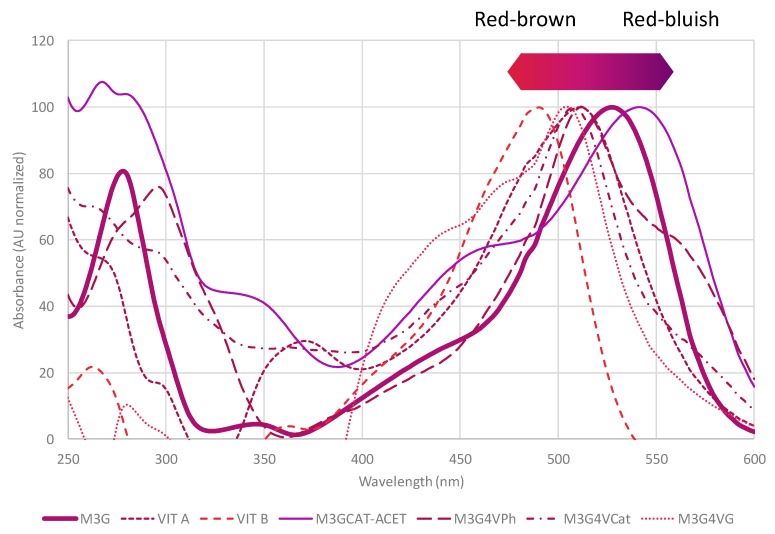
Normalized UV–Vis spectra of several pyranoanthocyanins and polymeric pigments. Tentative color of the range of red hue depending on the maximum of absorption in the visible spectrum (495–540 nm). M3G: malvidin-3-*O*-glucoside; VIT A: vitisin A; VIT B: Vitisin B; M3GCAT-ACET: malvidin-3-*O*-glucoside-ethyl-(+)-catechin; M3G4VPh: malvidin-3-*O*-glucoside-4-vinylphenol; M3G4VCat: malvidin-3-*O*-glucoside-4-vinylcatechol; M3G4VG: malvidin-3-*O*-glucoside-4-vinylguaiacol.

During fermentation, grape anthocyanins can be transformed into derived pigments by condensation reactions between them and various must compounds or metabolites produced by yeasts during fermentation. Pyranoanthocyanins are stable pigments formed during fermentation, and ageing following several reactions of electrophilic addition. They generally show a reddish-brown or reddish-orange color, their typical maximum being in the visible spectrum from 495 (vitisin B) to 515 (vitisin A) (Figure 3, Table 1). However, some of them can express red–bluish colors ranging from 538 to 583 nm; these pigments are known as portisins because they were first detected in Port wines [6,7]. Pyranoanthocyanin pigments are more stable under enological conditions because they are less sensitive to pH variations [26,27], resistant to SO_2_ bleaching by the saturation of carbon 4 [18] and have double the number of resonant forms because of the double pyran ring [3].

**Figure 4:**
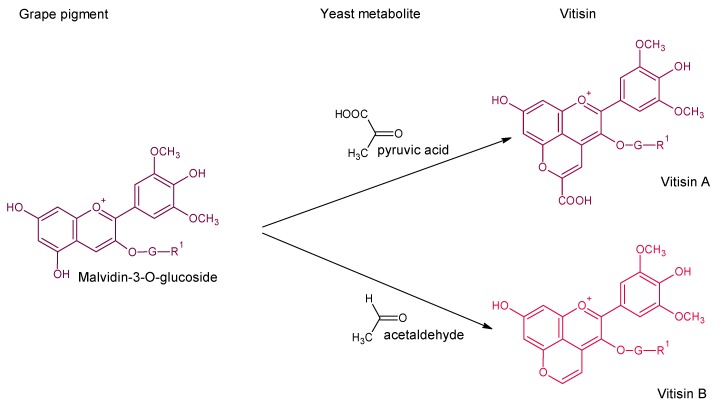
Vitisin formation from yeast metabolites. Main derivatives: vitisin A and B when grape anthocyanin is malvidin R^1^: –OH; acetyl vitisins when R^1^: acetic acid; *p*-coumaroyl vitisins when R^1^: *p*-coumaric acid. These reactions can also be produced from other grape anthocyanins (delphinidin, cyanidin, petunidin or peonidin).

Vitisins A and B were detected in wines [26] and their structures elucidated by LC-MS. Later, vitisin-type A and B derived from acetyl and p-coumaroyl anthocyanins were also found, mainly from malvidin, as they are the major grape acylated-anthocyanins, but also from other grape anthocyanin-3-*O*-glucosides [39,40,41,42]. The formation of vitisin-type compounds can be also favored by the addition of their precursors, acetaldehyde and pyruvate for vitisin B-type and vitisin A-type derivatives, respectively [43]. The formation of vitisins A and B during fermentation was observed as a consequence of the production of acetaldehyde and pyruvate by *Saccharomyces cerevisiae* (Figure 4), but there is also a strain dependence according to the production and releasing of these metabolites [29]. Therefore, it is possible to promote the fermentative formation of vitisins by using strains with higher pyruvate and/or acetaldehyde release. This biotechnology could help to increase color stability, especially in the long term, through the formation of stable vitisin pigments.

Maximum vitisin A formation has been observed at the beginning of fermentation (3rd–4th day) to correlate with the higher release of pyruvate. Towards the end of fermentation, yeast can reuse the released pyruvate when nutrient concentrations are scarce, and therefore, the rate of vitisin A production decreases [29]. It has been observed that the peak rate occurs when 57% of glucose is consumed [44], and most of the formation is done in the range of 20%–85% of glucose degradation. Formation of vitisin A continues after fermentation for several months while the precursors are available [44]. Concerning vitisin B, its precursor, acetaldehyde, is released continuously from the beginning to the end of fermentation, reaching the maximum formation of vitisin B at the end of fermentation [29]. High levels of vitisin A, but also some increments in the contents of vitisin B, have been observed in wines produced by carbonic maceration [45]. In these wines, total amounts higher than 15 mg/L were found. The peculiar pre-fermentative anoxic maceration increases the concentration of pyruvic acid in the musts, facilitating, subsequently, the condensation with grape malvidin yielding vitisin A.

When the formation of vitisin derivatives during fermentation is desired, the use of reduced doses of SO_2_ must be considered. SO_2_ behaves as a nucleophilic reagent and easily reacts with electrophilic molecules such as pyruvate or acetaldehyde [46], forming adducts and blocking them; thus, reducing the amount of these molecules able to produce vitisins with grape anthocyanins [36]. The pH can also affect the formation of vitisins. Higher concentrations of vitisins have been observed when the pH is 3.7 and lower values when it is lower or higher [36]. In addition, the fermentation temperature affects the formation of vitisin B because of the high volatility of acetaldehyde [47].

The use of non-*Saccharomyces* yeasts is another powerful bio-tool to promote the formation of pyranoanthocyanins. *Schizosaccharomyces pombe*, a non-conventional fission yeast [48], shows a peculiar metabolic pathway in which malic acid is degraded to ethanol and other secondary metabolites. This process is called maloalcoholic fermentation (MAF) and can be used as an alternative biological deacidification to malolactic fermentation in wines, using this yeast directly or reticulated in gel beads [49]. MAF involves the formation of pyruvate as an intermediate metabolite. It has been observed that *S. pombe* can release by this process, higher amounts of pyruvate than select *S. cerevisiae* strains [30,50]; thus, promoting the formation of large quantities of vitisin A-type derivatives during fermentation [30]. The main pyranoanthocyanin pigment observed was vitisin A, but acetyl vitisin A and *p*-coumaroyl vitisin A were also produced. The total amount of vitisin A-type derivatives found was always higher than with select *Saccharomyces*, ranging from double to quadruple. Some strains reached more than 11 mg/L compared to the 3 mg/L produced by *Saccharomyces cerevisiae* [30]. This higher formation of vitisin A derivatives by overproduction of pyruvate in *S. pombe* compared to *S. cerevisiae*, but also to other non-*Saccharomyces*, such as *Torulaspora delbrueckii*, *Saccharomycodes ludwigii*, *Lachancea thermotolerans* and *Metschnikowia pulcherrima*, has been verified in several studies [8,51,52,53]. Therefore, the use of *S. pombe* is an interesting and useful biotechnological tool to promote the formation of vitisin A-type derivatives during red must fermentation.

## 4. Vinylphenolic Pyranoanthocyanins

**Figure 5:**
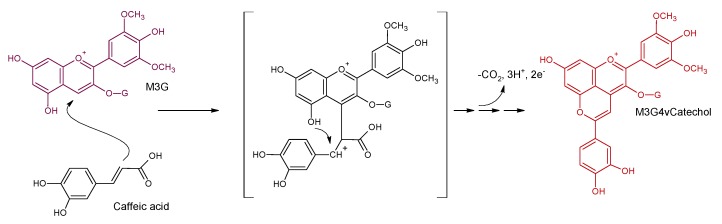
Chemical mechanism for the formation of malvidin-3-*O*-glucoside-4-vinylcatechol from malvidin-3-*O*-glucoside and caffeic acid during fermentation (adapted from [32]).

Vinylphenolic pyranoanthocyanins are stable pigments with similar properties to vitisins, also exhibiting the double pyran ring. They are formed during fermentation and ageing by reaction between hydroxycinnamic acids and grape anthocyanins. Initially, the formation of these derived pigments was described by a chemical route (Figure 5); it is a slow process with chemical condensation followed by structural reorganization involving oxidative conditions. The amount of vinylphenolic adducts formed increases along the ageing time, so these pyranoanthocyanins were suggested as age markers [32]. This route was described initially for the malvidin derivative of caffeic acid known as pinotin A (malvidin-3-*O*-glucoside-4-vinylcatechol), but it can be extrapolated to other cinnamic acids from grapes such as *p*-coumaric acid or ferulic acid, yielding, respectively, malvidin-3-*O*-glucoside-4-vinylphenol or malvidin-3-*O*-glucoside-4-vinylguayacol. Both compounds have been found in wines [31,34].

**Figure 6:**
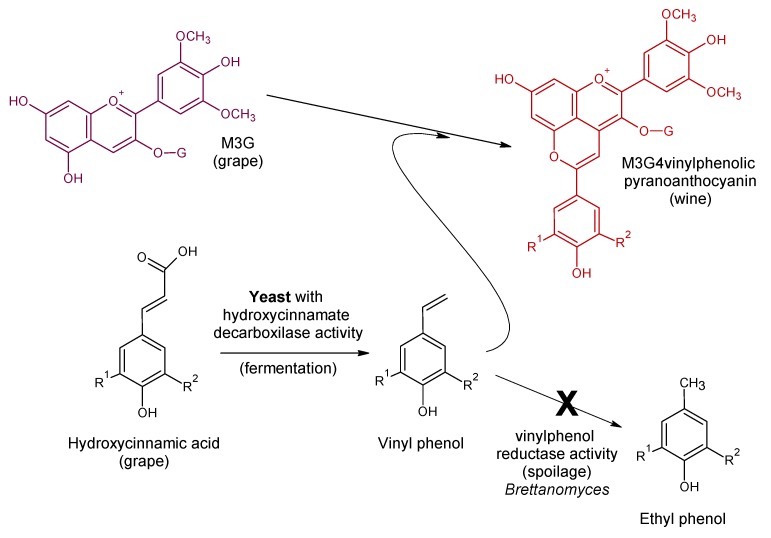
Biological-chemical mechanism for the formation of malvidin-3-*O*-glucoside-4-vinylphenol adducts from malvidin-3-*O*-glucoside during fermentation (adapted from [33]). Caffeic acid/M3G4vinylcatechol—R^1^: –H and R^2^: –OH; *p*-coumaric acid/M3G4vinylphenol—R^1^: –H and R^2^: –H; ferulic acid/M3G4vinylguaiacol—R^1^: –H and R^2^: –OCH_3_.

Later, it was observed that the use of *S. cerevisiae* yeast strains expressing hydroxycinnamate decarboxylase activity (HCDC), also known as phenolic acid decarboxylase (PAD), during fermentation, promotes the transformation of the hydroxycinnamic acids into their respective vinylphenols [54]. These molecules are highly reactive because of the vinyl group they have, developing a similar condensation reaction with grape anthocyanins [34] (Figure 6). The reaction is fast and occurs during the fermentation. This process was observed initially in sterile red musts fermented with HCDC^+^ *S. cerevisiae* strain with the production of malvidin-3-*O*-glucoside-4-vinylguaiacol, a compound that was not formed in the fermentations with the HCDC^-^ strain used as a negative control [3]. This mechanism is a mixed biological-chemical route and can be used as a biotechnological tool to promote the formation of stable vinylphenolic pyranoanthocyanins.

The formation of vinylphenolic derivatives of acylated anthocyanins (acetyl and *p*-coumaroyl derivatives) has also been observed during fermentation when HCDC^+^ strains were used and the must had suitable amounts of precursors [33]. Vinylphenol and vinylguaiacol adducts of acetyl and *p*-coumaroyl malvidin were observed in amounts lower than 1 mg/L. The same derivatives of non-acylated malvidin ranged from 5 to 12 mg/L. The formation of these vinylphenol adducts can be enhanced by the addition of the hydroxycinnamic acid precursors.

**Figure 7:**
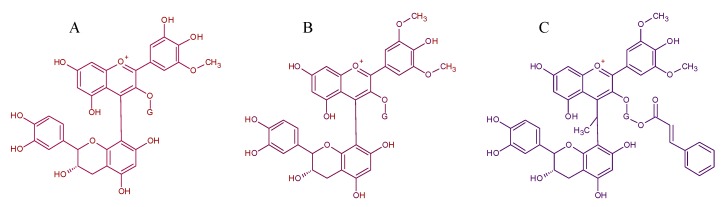
Proposed structures for pigment dimers found in Tempranillo microvinifications adapted from [8]. (**A**) petunidin-3-*O*-glucoside-4-(+)-catechin, (**B**) malvidin-3-*O*-glucoside-4-(+)-catechin and (**C**) malvidin-3-*O*-(6-*p*-coumaroyl)glucoside-4-ethyl-(+)-catechin.

Hydroxycinnamic acids are controversial molecules in wines because they are precursors of ethyl phenols, responsible for strong off-flavors produced in wines by the activity of the spoilage yeasts of the *Brettanomyces* and *Dekkera* genera. They have very low sensory thresholds, below 500 ppb [55], and produce unpleasant smells that are often described as “phenolic,” “leathery,” like “horse sweat,” like a “stable” or “varnish” [56]. The formation of these molecules normally occurs during barrel ageing, completely destroying the sensory quality of wines. However, when hydroxycinnamic acids are decarboxylated to ethyl phenols during fermentation by HCDC^+^ yeasts and chemically condensed with grape anthocyanins, they remain blocked in the pyranoanthocyanin. Therefore, they are no longer available to be reduced to ethylphenols by *Brettanomyces* (Figure 7). The formation of vinylphenolic pyranoanthocyanins during fermentation is a natural way of blocking the ethylphenol precursors by the formation of stable pyranoanthocyanin pigments. When 10 commercial HCDC^+^ yeast strains were used during fermentation to form vinylphenolic pyranoanthocyanins and the resulting wines were inoculated with 6-log CFU/mL of *Dekkera* in absence of SO_2_, the formation of ethylphenols was always lower or around the sensory threshold. Conversely, the control fermented with a HCDC^−^ yeast doubly exceeded the sensory threshold, reaching more than 1.1 mg/L of 4-ethylphenol [57]. Hydroxycinnamic acids in grapes are frequently esterified as tartaric esters. These esters can slowly hydrolyze during ageing, releasing hydroxycinnamic acids which can act as precursors of ethylphenols during barrel ageing if contaminated with *Brettanomyces*/*Dekkera*. The simultaneous use of cinnamyl esterases and HCDC^+^ yeasts produces the release of free hydroxycinnamic acids and their subsequent decarboxylations to vinylphenols that are susceptible to condensation with grape anthocyanins, forming vinylphenolic pyranoanthocyanins [57]. This biotechnology is an enzymatic, biological, chemical, natural way to form stable pigments during fermentation and simultaneously reduce the precursors of ethylphenol off-flavors.

The contribution of the vinylphenolic pyranoantochyanins, or the so called pinotins, to the formation of polymeric pigments has been assessed with an approach relatively similar to the quantification of the monomeric units found in condensed tannins. The quantification maker ions technique proposed by Laitila et al. [58] may contribute to the characterization of pinotin type oligomers in either proanthocyanidin and anthocyanin derivatives and adducts, respectively; anthocyanin derivatives; proanthocyanidin adducts; or ethyl mediated adducts formed during wine ageing. In this case, the marker ions used for the identification of pinotin oligomers in negative ionization mode UPLC-MS/MS were three malvidin-based pinotin structures formed by the condensation of malvidin glucoside with vinylphenol, vinylcatechol and vinylguaiacol. Yet, the presence of this type of oligomer is difficult to assess due to the low concentration in proportion to the other proanthocyanidin-malvidin derivative adducts.

## 5. Polymeric Pigments: Catechin Derivatives, Derivates by Acetaldehyde Bridge and Procyanidin Derivatives 

The formation of polymeric pigments in red wines is related to the presence of tannin structures available during the wine’s span life, but more specifically, to its aging [59] on one hand, and to the metabolism of fermentative yeasts on the other. Polymeric tannin structures and the influence they have in the stability of the colors of wines were already described after the second half of the 20th century. Polymeric pigments show less discoloration by sulfur dioxide and they are more stable to pH variations, as shown in experiments carried out at that time [59]. 

The interaction of anthocyanins with flavanol monomers or procyanidin units may take place by direct condensation or through ethyl linkages with acetaldehyde. Direct condensation is kinetically slower but it produces more stable adducts [60]. The nature of the anthocyanin may also play an important role in the kinetics of the reaction, being that cyanidin glucoside and petunidin glucoside are prone to condensing faster than malvidin glucoside [61]. This condensation mechanism is enhanced when there is lack of metabolites such as acetaldehyde in solution [62]. On the other hand, in the case of acetaldehyde mediated adducts, the reaction is faster in the acidic conditions observed in red wines but the stability of the oligomers formed is apparently weaker over time [63]. In this last case, the contribution of fermentative yeast strains may play an important role in the production of acetaldehyde.

The influence of different yeast species and strains in the formation of oligomeric pigments in red wines has been reported in the past two decades [3,8,30,53,60,64,65]. The feasibility that (+)-catechin and procyanidin B2 have to condense anthocyanins during fermentation with different fermentative species was observed in Tempranillo fresh musts from Ribera del Duero in Northern Spain [8]. Non-*Saccharomyces* species such as *Schizosaccharomyces pombe*, *Torulaspora delbrueckii* and *Saccharomycodes ludwigii* reduced the number of dimers of malvidin glucoside condensed directly with (+)-catechin with respect to *Saccharomyces cerevisiae* strains. Nonetheless, the fermentations carried out with these non-*Saccharomyces* strains allowed the condensation of (+)-catechin with other anthocyanins and also mediated with acetaldehyde. The molecular structure of these oligomeric pigments is given in Figure 7. 

Although some *Saccharomyces cerevisiae* strains have reported elevated concentrations of acetaldehyde production, other non-*Saccharomyces* yeasts are also able to produce this by-product in such concentrations [66]. Among these species, in the early stages of the fermentation is possible to find *Kloeckera apiculata*, *Candida stellata* and, towards the end of the fermentation and together with *Saccharomyces cerevisiae*, the species *Zygosaccharomyces fermentati* also has phenotypes producing large amounts of acetaldehyde (up to 67.5 mg/L). The presence of acetaldehyde in wine during ageing promotes the formation of ethyl-linked oligomers [63]. According to Rivas-Gonzalo et al. [67], the acetaldehyde reacts with the catechin in acidic media before it can react with any anthocyanin moiety. The formation of a carbocation in the acetaldehyde after it condenses with the catechin monomer is needed for the condensation with the anthocyanins later. 

**Figure 8:**
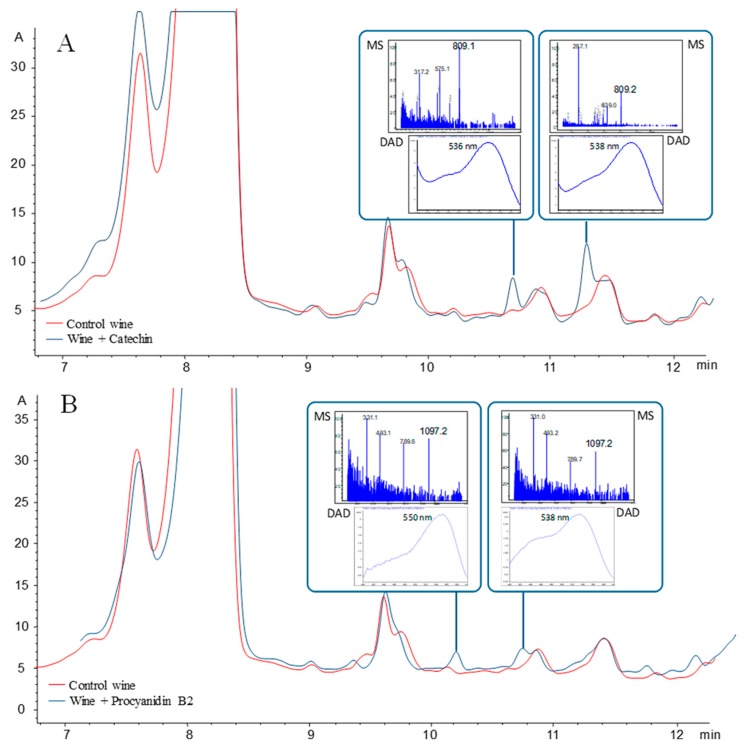
Fragment of the chromatograph of experimental wine enriched with (**A**) (+)-catechin and (**B**) procyanidin B2. Two peaks were observed for the dimer of malvidin glucoside with catechin in the first case and two peaks for the trimer of malvidin glucoside with procyanidin B2 in the second. Molecular ions and fragments (*m*/*z*) are shown in MS spectra and λmax is shown in the diode-array detection (DAD) signal for each of the selected peaks [53].

Unlike vitisins or pyranoanthocyanins with lower maximum absorption wavelengths (Table 1), the color observed in oligomeric pigments has a shift in the maximum wavelength towards lower frequency wavelengths, as shown in Figure 8 for two oligomeric pigments observed with HPLC-DAD/MS in experimental wines. Dimeric malvidin-3-*O*-gucloside-4-ethyl-catechin and malvidin-3-*O*-glucoside-4-ethyl-procyanidinB have molecular ions [M]+ 809 with λmax 536–538 nm and [M]+ 1097 with λmax 538–550 nm, respectively.

Larger molecular weight flavanol oligomeric copigments have been found and described in red wines with up to seven monomeric units: dimeric oligomers with catechin and epicatechin monomers as components [68,69]; dimeric structures esterified by gallic acid units [69,70]; trimers and tetramers with and without esterification by gallic acid [69,71]; and, lastly, pentamers to heptamers with doubly charged ions [72]. These relatively short chain tannins may have the ability to condense anthocyanin moieties to form more stable pigments during the ageing processes of red wines. As soon as short chain tannins continue to condensate with catechin monomers through ethyl bridges (CH-CH_3_), polymers aggregate into colloidal size particles. This phenomenon may be responsible for larger polymeric structures precipitating [73].

## 6. Anthocyanin Adsorption in Yeast Cell Walls

Yeast can develop a high external cell wall surface, easily reaching 10 m^2^/L of must during fermentation [10]. The amount of color adsorbed depends not only on the structure and polarity of anthocyanins [9,74], but on the structural state of the cell wall [75]. Even when there is not a clear evidence of what the binding molecules are, it is quite logical to think that the external surface of the cell wall, formed mainly by globular mannoproteins, has a key role in the anthocyanin adsorption, and these mannoproteins are probably responsible for the retention. Evidence of the interaction between mannoproteins and phenols and the lack of interaction between β-glucans and phenols has been observed with microscopy [76]; this interaction seems stronger between phenols and mannoproteins from whole cells than with cell wall fractions. Complementary evidence, such as the possible effect on color stability of polysaccharides released by yeast, support this theory [77,78]. A selective retention of anthocyanins depending on the *S. cerevisiae* yeast strain used for fermentation has also been observed [10], ranging from 2% to 6% for total anthocyanin content, but from 7% to 30% for some acylated anthocyanins. This adsorption can have a strong impact on wine color by the quantity of pigment removed [79], and therefore, on wine’s sensory quality [80], since color is the first perception when tasting wine in a glass. The selection of strains/species of yeasts with low anthocyanin adsorption is an interesting way to reduce the losses of color during winemaking [81], especially useful in wines from grape varieties with low anthocyanin contents or in regions in which anthocyanin synthesis is reduced because of climatic conditions. Differences in the amounts of adsorbed anthocyanins have also been observed among different *Saccharomyces* and non-*Saccharomyces* species [3].

## 7. Stable Pyranoanthocyanins and Ageing on Lees

Ageing on lees (AOL) is the technique in which the wines are stored and matured with all or some of the yeast cells that conducted the fermentation [82], but it can also be done with the addition of an external biomass produced in a fermenter with some specific yeast [12]. This process increases mouthfeel and structure due to the release of cell wall polysaccharides/mannoproteins, but also enhances the aroma and has some repercussions in phenolic compounds and pigments. The use of emerging technologies as ultrasounds, ultra-high pressure homogenization or microwaves, speed up the cell lysis, and therefore, the release of these molecules [83,84,85,86,87,88,89].

The role of yeast mannoproteins on the protective effect on wine pigments is already being discussed in literature; some authors have observed the higher stability of wine color in wines aged on lees (AOL) or with added commercial mannoproteins [77,78,90]. This could be supported by the potential interactions in colloidal dispersion between mannoproteins/polysaccharides and anthocyanins, and also by the antioxidant protection of reductive nitrogen compounds released during ageing on lees, such as glutathione and others. However, other researchers studying commercial mannoproteins have opposed opinions regarding the effects of these molecules on the colloidal stability of wine pigments and the repercussions in color stability [91,92].

When comparing the evolution of grape pigments as malvidin-3-*O*-glucoside with several pyranoanthocyanins (vitisins and vinylphenolic) during the AOL of red wines with *S. cerevisiae* and several non-*Saccharomyces* yeasts, it was observed that M3G decreases by 85% in 3 months; however, the pyranoanthocyanins’ content was reduced by 30%–40% of the initial concentration [93]. When several *Saccharomyces* strains were studied for more than one-year, low reductions in vitisins were observed (10%) compared to a strong decrease in M3G (50%) [94]. Similar effects were observed during a 6-month AOL with several selected strains of *S. cerevisiae* [12]. Moreover, vinylphenolic pigments increased their concentrations, and over time the formation of some of them that were not detected in the initial wine, was observed. The use of ultrasounds affects the pigment content in general by reducing the final concentrations, but specifically it also decreases the content of vitisins and vinylphenolic pyranoanthocyanins [85,86].

## 8. Emerging Techniques to Better Implant Non-*Saccharomyces* Yeasts during Fermentation

The use of non-*Saccharomyces* yeasts can facilitate the formation of stable pigments during fermentation; however, most of them have implantation difficulties because they are less competitive than *S. cerevisiae* for must fermentation. This is especially remarkable when crushed grapes are fermented, as is the case of red winemaking with all the wild, indigenous yeasts usually found on the external pruines of the skins. Non-*Saccharomyces* usually have lower fermentative power than *S. cerevisiae* and most of the species described are able to ferment from 1% to 9% vol. of ethanol, but subsequently, ethanol inhibits their development. Some non-*Saccharomyces* have a fermentative power similar to that of *S. cerevisiae*, reaching alcoholic degrees of 13%–15% vol. depending on the strains, as is the case for *S. pombe* [48,95]; however, its low fermentative rate [48] makes them often unable to compete with the faster *S. cerevisiae*. The use of new non-thermal emerging technologies, such as high hydrostatic pressure (HHP) [96,97], ultra high pressure homogenization (UHPH) [98], pulsed electric fields (PEF) [99,100], ultrasonication (US) [101], pulsed light (PL) [102] and β-irradiation (βi) [103], helps to eliminate the wild indigenous grape microbiome, facilitating the implantation of non-*Saccharomyces* yeasts and also promoting the extraction of phenols, and anthocyanins especially [104,105,106]. Some emerging techniques such as HHP, have shown the potential to increase the formation of stable pyranoanthocyanins such as vitisin A, but also to promote anthocyanin polymerization at extreme pressures [20,107]. US treatment at 100 W, 40 min has also increased the yield of methylpyranocyanidin-3-*O*-glucoside by 32.5% [108].

Some of these technologies have some advantages for industrial scale-up and cheaper applications. UHPH, PEF, US, PL and βi can be applied continuously; however, HHP is a discontinuous or batch process [105]. The devices to treat by HHP, UHPH, US and βi have a cost ranging from 1 to several million €; PL is a cheaper technology, ranging from 0.1 to 0.05 million € depending on the treatment dose and volume to be processed. UHPH needs a particle size of less than 0.5 mm to be used [98], so it cannot be applied on whole or crushed grapes; only the must can be processed. PL produces a surface effect because light radiation has a low depth of treatment, lower than 1 mm; therefore, mainly affecting grape skins, and destroying whatever microorganisms are present, including yeasts, bacteria and spores. The effectiveness of HHP, PEF, and especially, US on bacteria and spores, is limited. Therefore, PL is a cheap and continuous technology useful to remove most of the microorganisms from grapes’ surfaces. In addition, it does not affect grape composition, especially preserving those molecules with sensory impacts, such as aromas or pigments.

## 9. Conclusions

Pyranoanthocyanins and polymeric pigments are the most stable forms of color under enological conditions; therefore, by favoring the formation of these compounds it is possible to achieve a better and more stable color. The use of selected yeasts strains of *S. cerevisiae* or specific non-*Saccharomyces* species can favor the formation of stable vitisins, vinylphenolic pyranoanthocyanins and polymeric pigments in a natural way during fermentation; therefore, contributing to a more balanced and durable color.

## Figures and Tables

**Table 1:** Main derived pigment formed in wines by the fermentative action of yeasts.

Pigment	Type	Structure and Tentative Color	Visible λ max. (nm)	*m*/*z* [M − H]^+^	*m*/*z* aglycone	Tentative RGB Color	Promoter Yeast Species	Reference
**Monomeric**								
Malvidin3-*O*-glucoside	Anthocyanin	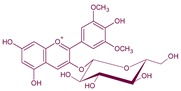	528	493	331	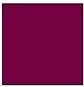	From grape	-
Vitisin B	Pyranoanthocyanin-acetaldehyde derivative	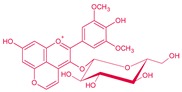	495	517	355	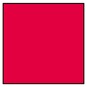	*S. cerevisiae*	[26,28,29]
Vitisin A	Pyranoanthocyanin-pyruvate derivative	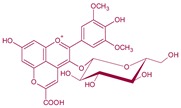	515	561	399	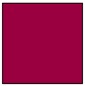	*S. cerevisiae*, *S. pombe*,	[26,28,29,30]
Malvidin-3-*O*-glucoside-4-vinylcatechol	Vinylphenolic pyranoanthocyanin-caffeic acid derivative	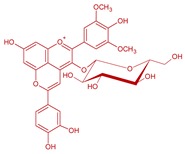	504	625	463	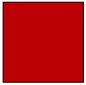	*S. cerevisiae*, *S. pombe*	[31,32,33]
Malvidin-3-*O*-glucoside-4-vinylphenol	Vinylphenolic pyranoanthocyanin -*p*-coumaric acid derivative	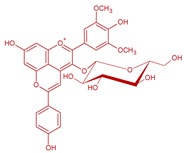	504	609	447	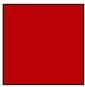	*S. cerevisiae*, *S. pombe*	[30,31,33,34,35]
Malvidin-3-*O*-(6-acetyl)-glucoside-4-vinylphenol	Vinylphenolic pyranoanthocyanin -*p*-coumaric acid derivative		508	651	447		*S. cerevisiae*, *S. pombe*	[30,31,33]
Malvidin-3-*O*-(6-p-coumaroyl)-glucoside-4-vinylphenol	Vinylphenolic pyranoanthocyanin -*p*-coumaric acid derivative		508	755	447		*S. cerevisiae*, *S. pombe*	[30,31,33]
Malvidin-3-*O*-glucoside-4-vinylguaiacol	Vinylphenolic pyranoanthocyanin -ferulic acid derivative	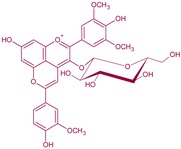	512	639	477	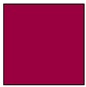	*S. cerevisiae*, *S. pombe*	[30,31,33,36]
Malvidin-3-*O*-(6-acetyl)-glucoside-4-vinylguaiacol	Vinylphenolic pyranoanthocyanin -ferulic acid derivative		520	681	477		*S. cerevisiae*, *S. pombe*	[30,33,37,38]
Malvidin-3-*O*-(6-p-coumaroyl)-glucoside-4-vinylguaiacol	Vinylphenolic pyranoanthocyanin -ferulic acid derivative		522	785	477		*S. cerevisiae*, *S. pombe*	[30,33,37,38]
**Polymeric**								
Portisins	Vinylphenolic pyranoanthocyanin – phenol, catechin or procyanidin derivative in R2Glucose, acetylglucose or p-coumaroylglucose in R1	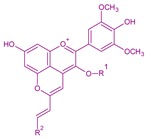	Phenol:538Catechin:572Procyanidin:583			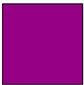		[6,7]
